# An Inquiry into the Moral, Social, and Intellectual Condition of the Industrious Classes of Sheffield. Part 1.—The Abuses and Evils of Charity, Especially of Medical Charitable Institutions

**Published:** 1839

**Authors:** 


An Inquiry into the Moral, Social, and Intellectual Condition of the Industrious Classes of Sheffield. Part I.—The Abuses and Evils of Charity, especially of Medical Charitable Institutions. London. Longman and Co. 1839. 8vo. pp. 132.

The plan of this work comprises a number of very important subjects in morals and social politics, we give them in the author's own words.

"I. We propose to examine the condition of the charitable institutions of the town; to point out their beneficial and injurious tendencies, and to suggest, in regard to some of them, such modifications as shall increase the good and diminish the evil with which they are fraught. II. We shall investigate the social, intellectual, and moral conditions of the working classes. III. The effects of unions or combinations of workmen, on themselves and on trade. IV. The educational institutions of the town. V. The number, strength, and wealth of sick clubs, and the causes of their frequent inefficiency or insolvency. VI. The consumption of malt liquor and spirits in the town, and the effects of beer-houses on the morals of society. VII. The amount and character of crime for the last five years. VIII. The effects of the poor laws on society. IX. The character of the debts sought to be recovered in the Court of Requests, and of those which are served out in gaol. X. The effects of the manufactures of this town on the character and condition of workmen compared with those of other towns where machinery is extensively employed."

The part now published and under review is chiefly occupied with the medical charities of the town, a subject with which the author's situation as medical officer to both the infirmary and dispensary has given him an opportunity of becoming well acquainted. The work evinces much originality of thought and patient and accurate observation, and is written in a pleasing and elegant style, as will be shewn in the extracts we shall have occasion to make from it.

The volume is opened with some remarks on the state of society in Sheffield; concerning the trade, we are told—

"It is exceedingly unlike in its character, and consequently different in its effects, from that which employs the genius and enterprise of Manchester, Leeds, or Nottingham. It stands indeed alone. The statistics of these important towns would throw little light on the constitution of society here. With us the distinctions between master and man are not always well marked. Persons are to a great extent both. The transition from one to the other is easy and frequent in those branches where the tools are few and simple, and the capital required extremely small, which applies largely to the whole of the cutlery department. This circumstance produces peculiar and striking effects on the character and condition of the working classes.

Most of the manufacturing towns in the kingdom have undergone extraordinary changes within the last half century. Manchester, Glasgow, Birmingham and Leeds have, within this period, more than trebled their population and capital, and are now rapidly progressing. Imagination is not daring enough to define the probable limits of their increase. Genius, enterprise, and industry do not admit of severe calculation.

Sheffield has also trebled her population and wealth. She has not, however, taken her proper position among first-rate towns. Her intelligence and wealth are by no means justly estimated. This arises in a great measure from the nature of her productions. Her merchants and manufacturers cannot become princes. There is not sufficient play for large fortunes, nor would these secure those peculiar advantages which invariably mark their influence in other situations. Novel and ingenious inventions, extensive speculations in the purchasing of raw material, are not features characteristic of our manufactures. The making of fortunes is with us a slow process. It is, however, far from being partial, nor is it unaccompanied with advantages, which are often only incidental attendants on the sudden accumulation of wealth.

If our fortunes individually are much less than those of the merchants of Manchester or Glasgow, the longer period in the making of them allows the mind time to adapt itself to its improved circumstances—not merely the speculative and money-getting part of the understanding, but the whole of its social, moral, and intellectual powers, without which riches are a questionable good under this change. Accordingly, wealth and intelligence are with us more generally associated than in towns where immense fortunes are rapidly made."

Speaking of the ignorance and dissipation of the lower orders, the author admits that knowledge is unquestionably the great remedy for these evils, and says, that it is not to be inferred that he is opposed to education or despairs altogether of some good being effected by it; that he has ever been friendly to the diffusion of knowledge, and regards it as the handmaid to every virtue; that his present wish is not to abridge, but if possible to extend its blessings; and that the present enquiry is, indeed, the prompting of that feeling; but that he differs from the zealous advocate of educational systems with respect to the means it is desirable to employ, and the results to which we may confidently look forward. Writers on education, he says, belong to the Martin school of painting. They study effect, but are not practically acquainted with the materials they fashion into beauty and adorn with eloquence. The laws of Nature, on which they pretend to found their systems, will offer insurmountable obstacles. Manufactures and commerce spring from these laws and are in strict harmony with them, and the wealth, distinction, and luxurious enjoyment arising from these will always be motives to action. He says that habits engendered bp a palmy state of trade are not corrected by depression. That the young of both sexes are forced early to work, and when thrown out of employment they are with few exceptions disposers of their own time, and that then—

" The temptations are not towards intellectual pursuits or moral obligations. The path in which these lie is too straight and restrictive in its privileges. The indulgence of the passions opens a much wider and more attractive field. Is this the school proper for the future mother? Is it here that the systems of education will reach her, and convert all her sensual tendencies into virtuous emotions, and her grovelling views into high minded and enobling principles? The systems may play around her head, but they will make little impression on her understanding. A return to prosperity will cause her to retrace her former steps, perhaps with less virtue and fewer good intentions.

These changes and habits are the inevitable fruits of manufactures. The hand of the dyer will ever be imbued with the colour in which he works."

The author of the work before us cannot agree that the beneficial working of the parochial schools in Scotland, and the comprehensive system of education adopted in Prussia, is evidence of the necessity and excellence of a national system of education in this country. He says, in Scotland, where the operation of the schools is efficient, it is in agricultural or thinly populated districts, where wages are low and subject to little variation. That the more agricultural the occupation, and the steadier the remuneration, the more intense and elevated will be the moral and religious feelings. He says there is nothing in common between Prussia and this country. Prussia possesses only a graduated freedom, and its resources are sufficient for its wants. The situation of the people in that country is enviable, but unfortunately it is beyond imitation.

A national system of education cannot be urged on the plea that the mass generally are too poor to provide for their own mental improvement; for one-fifth of what they spend in dissipation would be an ample fund for the purpose. Education cannot possibly be otherwise than imperfect with the great mass who have to labour for the necessaries of life; whose bodily exertions offer a sufficient outlet for the escape of nervous energy. It is incompatible with the laws of the animal economy, that both mind and body shall toil, and knowledge does not fall upon the understanding like the sunshine upon the flower. That amongst the working classes, at some future time, the duties of the day will be so divided, that bodily and mental exertion will alternate in such a manner that the one will be a stimulus to the other, rather than a process of exhaustion; he says, is not impossible, but is about as probable, as that wings will at the same time shoot from the shoulders, to enable the artizan to breathe a purer air during his intellectual flights. Knowledge is as prone, he considers, to give rise to pride, vanity, and conceit, as to create self-respect or becoming independence. The predominating passion will be determined by the natural constitution of the mind, and the circumstances in which the individual is placed, rather than by the amount of knowledge possessed. He tells us there never was a period when knowledge was so generally diffused as now, and there never was an equal amount of vice, dissipation, or reckless conduct.

Now we cannot help thinking that this is looking too much on the dark side of human nature. We see no reason why an enlightened system of education should not perform as great an amount of good in a manufacturing, as in any other population. The fluctuations in wages arising from changes of commerce, must always have an ill effect on the morals and happiness of the people; but education is only the more necessary on this account, as being the best means of enabling them to provide against adversity, and conduct themselves well under prosperity. We consider that the vice and dissipation amongst the manufacturing population has arisen mainly from their pecuniary resources far outstripping their mental cultivation; and one other great cause is the influx of people from all parts of the kingdom to the manufacturing districts, a great portion of whom would necessarily be the worst characters in society, as these are the first to move away from their homes. But we cannot expect this influx to the manufacturing districts to continue in the same ratio. Our author himself remarks, that the strikes amongst the workmen of this country, have thrown our continental neighbours on their own resources—and if this influx should continue, what so likely to stay the evils of it as a comprehensive system of education which will humanize the population throughout the empire, and will put the agricultural labourers themselves in such a condition that, should they remove amongst manufacturers and receive high wages, they will not break out into all manner of excesses. We see nothing in the occupation of the manufacturer incompatible with order and morality, and, indeed, we have personally known large bodies of the manufacturing population, when they were not in large towns, and were living amongst their own relatives on the spot where they were born, and where their parents had lived and laboured before them—we have known those people to be equally sober and religious, and more intelligent than the agricultural population in any part of the country.

We see no reason why the salutary restriction of labour in childhood, which has already infinitely benefitted the health of the rising generation amongst the cotton and linen factories, may not be extended to other branches of manufacture, with benefit to health, and, joined with a proper system of education, to morals also. We admit that knowledge does not always produce a moral or dignified character, and that the predominating passion will be determined by the natural constitution of the mind, and the circumstances in which an individual is placed, but the natural constitution of the mind is the same, on an average, in all classes of society, yet the majority of the upper and middle classes are sober and respectable, though surrounded by the very objects that afford temptations to the lower orders. Therefore we doubt not that the same intelligence which has put out of countenance the inordinate consumption of wine, once fashionable in the highest ranks of society, will, ere long, have a similar effect on the gin and whiskey of the lower orders. We have been led to conclude, from our own observation and the unanimous testimony of others, that there has been a great decrease of crime and vice of late years, and if this is not the case in Sheffield, that town is a remarkable exception to the rest of the country. Surely cock-fighting, dog-fighting, badger-baiting, pugilism, excessive drunkenness, and chewing tobacco, are not so common there now as thirty years ago, and we should expect that the most indecent songs do not continue to be sung publicly in the streets, and that there is a notable increase of comfort in the homes of the working people.

The measures the author would adopt for ameliorating the condition of the working classes, will be seen in the following quotation, on which we will not comment at present.

"The mass will not be taught, however, to depend on their own exertions by any enlarged views presented to the mind. It must be by making them feel the effects of misconduct and intemperance, and they will never feel them acutely so long as their necessities, and those of their family, are promptly relieved, without regard to the previous earnings or conduct of the individuals. The more that is done for them, the less they will certainly do for themselves. The awakening of their own faculties, or the breathing process, by which the mind starts into conscious existence and vigorous exercise, will mainly be accomplished by throwing them on their own resources. They must not be taught to fall back upon charity, in cases of trivial emergency, as a good to which they have an undoubted right. This feeling takes away all idea of exertion. The sacrifice of independence should not be made without a struggle. The effort to preserve it is salutary, and ought to be encouraged. Supposing, in the attempt, debts and obligations are incurred which compel strict economy, or occasion certain privations. There is nothing hard, cruel, or unjust in this. There would be great cruelty and injustice were it otherwise."

The truth of the following observations will be at once perceived.

"Let the charity, whatever be its nature, hinge on circumstances. To present it as a certainty to all who may please to apply, without the strictest inquiry into the condition, habits, and income of the parties, is to encourage the most flagrant impositions; is, in fact, to hold out a premium to idleness, dissipation and intemperance. Charity should never be on an inclined plane. The path leading to it should have difficulties, but of such a nature, that they neither arrest the efforts, nor for a moment fret the honest feelings of the really necessitous. The indiscriminate exercise of it is the parent of many evils. It is often absorbed not only by improper objects, but the stream, in consequence, does not flow in sufficient abundance to the truly miserable and destitute."

The author then proceeds to investigate the working of the medical charities of Sheffield, and says, that if a thorough searching reform be not carried into effect in them, they will go far towards pauperizing the whole of the working classes. He says, "It is indeed a fact, placed beyond all question, that any number of tickets may be procured for the Sheffield General Infirmary, without the applicants, or their condition, being in the slightest degree known to the parties who recommend. It is not necessary, in this place, to explain how,—the fact and the means are equally notorious. One individual, whose annual subscription is two guineas, recommends to the infirmary almost one-third of the patients, without knowing anything of their circumstances or condition in life." This institution, he says, when first established was for the poor and necessitous of all nations; and this was understood to mean, such only as were incapable of otherwise procuring the required aid; but that now the artizan never dreams of the possibility of rejection on the ground of being in full and regular employment, and being amply remunerated for his labour.

The following are some of the conditions which, in the opinion of the author, ought to exclude from a participation in the charity. "*In the first place.* Single men in employment have not the slightest claim. *Secondly.* It could not possibly be contemplated to extend the charity to the married operative in work, with only a young and small family. *Thirdly.* The operative receiving high wages, if he please to work, though he may have several children, can have no just claim on the charity. *Fourthly.* The operative who has several sons or apprentices working for him, is in no degree entitled to the benefits of the charity. *Fifthly.* Neither male nor female servants in place, and attending to their respective duties, are proper objects of relief." Before bringing forward facts to show that these conditions have been by no means attended to, the author adduces some general considerations to show that the charity is greatly imposed upon. He says, the distresses of the community must bear a strict relation to the state of trade; there must be more distress when many hands are thrown out of employment, than when labour is plentiful and wages high. Misery cannot be a fixed quantity, always floating in society; and yet, if the registered demand for charity be any criterion, there is scarcely any variation.

"From Midsummer 1835, to Midsummer 1836, between which periods trade was better in this town than it had been known for years, the number of patients admitted on the books of the Infirmary was 3,126. From Midsummer 1836 to Midsummer 1837, between which periods the trade was exceedingly depressed, the number was 3,431, being an increase only of 305 patients. Between the former periods the number of patients on the books of the Dispensary was 2,888. Between the latter periods, that is, from July 1836 to July 1837, the number was 2,575, being less by 313 patients. According to these returns, there were eight patients more, during a prosperous state of trade, recipients of medical charity, than during the severe depression of it."

He says, he believes the demand for charity will always be equal to the means of supply. It is then shewn by numerical tables, constructed from the reports of the Infirmary, that above one-fourth of the patients annually benefitted, think it too much trouble to return thanks, and that this number is greatly on the increase. It is also shewn by tables that irregularities amongst the in-patients are also on the increase. From Midsummer 1822 to Midsummer 1827, one patient in 49 was discharged for irregularity, and from Midsummer 1833 to Midsummer 1838, one in every twelve. This is considered another proof of the relief of improper objects, as the truly necessitous are always thankful. Great numbers of Irish labourers and tramps are admitted, who require food, clothing, and rest, more than physic, and ought to be relieved by the parish. Patients are often conveyed to the Infirmary in hackney coaches, not from inability to walk. Females visit the institution in elegant cloaks, shawls, and clogs, and often for very trifling complaints, and for the removal of disease which just perceptibly mars the beauty of the face or neck.

The following is an account of a hundred patients taken indiscriminately on each day of admission, for several weeks at the Infirmary.

| | | |
|---|---|---|
| " 1. | Single men past the age of twenty-one | 15 |
| 2. | Persons married but without family | 11 |
| 3. | Persons having from one to two children | 32 |
| 4. | Persons having three children | 6 |
| 5. | Servants in place | 7 |
| 6. | Persons having four children | 10 |
| 7. | Widows | 7 |
| 8. | Single women, in no permanent situation | 2 |
| 9. | Persons having five or six children | 4 |
| 10. | Apprentices | 2 |
| 11. | Persons having seven or eight children | 2 |
| 12. | Widowers | 2 |
| | | 100" |

The author says, if the single men have been ill long and have exhausted their resources; or if they have had no employment for a considerable period, they are proper objects. In thirteen out of the above fifteen cases, however, it was found on inquiry to be quite otherwise. Of these thirteen, " nearly two-thirds were admitted into the house, and some of the others made application, assigning as a reason, that they would be much more comfortable than at home. Several were members of sick-clubs, and had given notice of their situation, so that whilst they were maintained by the charity, and afforded every proper indulgence, they were at the same time receiving ten shillings a week from the club." The private circumstances of the remainder of the hundred patients are inquired into, and the majority are found not to be proper objects of charity. The widows and women in no permanent situation, are considered true objects of charity. Some of the people having a number of children, were in needy circumstances; others not so, their children being grown up and receiving wages. The maid-servants and apprentices are considered the most objectionable item of all, as their claim is upon their employers, and it is only when they become unequal to their duties and are thrown upon their friends or their own limited resources, that they become entitled to relief.

At the time the author was writing, the infirmary contained 77 in-patients, of whom he gives the following list.

| | |
|---|---|
| “In the first place, single men | 19 |
| 2ndly, Single women | 6 |
| 3rdly, Persons married but without family | 11 |
| 4thly, Men married, and patients with one or two children | 5 |
| 5thly, Women married, and patients with one or two children | 5 |
| 6thly, Persons married, with three children | 5 |
| 7thly, Persons married, with four children | 3 |
| 8thly, Persons married, with five or six children | 2 |
| 9thly, Persons married, with seven children | 1 |
| 10thly, Widows | 4 |
| 11thly, Widowers | 5 |
| 12thly, Apprentices | 3 |
| 13thly, Children patients | 7 |
| Total | 77” |

The occupations of all the men are enumerated, and it is concluded that two-thirds of the 77 patients were by no means in adverse circumstances, and that several of the others were proper objects for the poor-house. We extract the following.

“There is another fact much more important, and deserving the most serious consideration, namely, *the rapidly increasing number of Irish applicants.* We do not allude to them as improper objects. The fact is valuable in this relation, but much more in connexion with the condition of their country, which, if permitted much longer to continue, will extend its demoralizing and pauperizing effects throughout the whole of England. To the consideration of this interesting subject we shall return. Ten of the forty-six adult male patients are Irish.”

Respecting the funds of the Infirmary we are told,

“For several years past the annual subscriptions and landed property of the Infirmary have been found altogether inadequate to cover the current expenses of the establishment. The deficiencies have been met by frequent and handsome legacies, but which being incidental and not permanent resources cannot possibly be calculated upon for the future. If they fail, one of two results is inevitable, the gradual absorption of the funds, or a diminution of the usefulness of the charity proportionate to its limited means. It is questionable whether both be evils. The latter would indeed be a serious one; it does not, however, necessarily follow from the former. *Charities are seldom liable ta great abuses when depending on the sympathies and liberality of contemporary benevolence.*”

The author then gives an account of the other medical charity in Sheffield, namely the Dispensary, illustrated also by statistical tables, by which it appears that this institution is as much imposed on by improper objects as the infirmary, and that the ingratitude of the patients is greater, for not one in three takes the trouble to return thanks.

He considers, and we think justly, that this indiscriminate charity is attended with great evils to society.

" This departure from the rules is accompanied with a moral effect, of greater importance to society than the misapplication of the funds. As already stated, by such conduct we are teaching the first injurious lesson of asking charity. We are breaking down the bulwarks not only of independence and a proper feeling of pride, but, indeed, the outposts of virtue itself. The mind is trained to look down, and this is always at the expense of those fine and delicate perceptions of propriety, which cannot be too carefully cultivated. The precincts of a charity have a deadening influence on its numerous and hungry expectants. Physical evil may be alleviated, but the high-toned morality of the mind is lowered."

The board room of the infirmary is very ill attended, and on an average two out of every three of the recommendations to both institutions are given by persons to whom the circumstances of the applicants are almost entirely unknown. For the reform of these abuses, the author suggests the adoption of the following printed form of recommendation, by which the recommender would be compelled to analyse the circumstances of the applicant.

1. Name, age, and residence.
2. Single, or married. If married, what family under nine years of age?
3. Trade of the husband, father, or patient?
4. For whom does he work?
5. Name of the recommender.
6. The disease.

And as a further protection against imposition he recommends the weekly publication of these particulars in a tabulated form, in the newspapers of the town. Leaving out, however, the name of the disease.

We entirely agree that the distribution of charity to every one who may ask is a great evil, and exerts a demoralizing influence, and that the circumstances of the applicants ought in every case to be enquired into; and we think the printed form of recommendation proposed by the author, a valuable means of promoting this inquiry. However, we cannot concur to the full extent in the stringent measures advised in this work. Though a person may have no children, or only one or two, we do not agree that he is always an improper object, before long illness or want of employment has reduced him to a state of entire destitution; there is charity in preventing misery, as great as in relieving it. And if a man has been intemperate and extravagant, we are not amongst those who think extreme adversity the best school for teaching morality; we do not say, let him and his family feel the effects of pinching want. Illness is a severe affliction of itself. And especially we cannot agree that it is desirable debts should be contracted. When a person is ill he may die, and the reflection that he is buying that for which he can never pay, is not calculated to strengthen his morality or comfort his last hours. If an unmarried man, he is probably lodging amongst strangers, as numbers are in every large town, and what sort of attendance and watching can he expect for the promise, that if he recover he will repay those around him? Although extreme profusion in charity is calculated to undermine the spirit of independence amongst the working classes, extreme niggardliness will not encourage it; unless, indeed, that kind of independence which would lead them to hustle respectable people from the pavement, as they pass them in the street. We do not think the subscribers to medical charities will consent to exhibit the names of those benefitted in the newspapers, nor do we think they ought. The feeling of self-respect which the author justly considers so important, is intimately blended with the desire for the respect of others, so that, though this measure might deter many people from applying for charity, we doubt its good effect on those who would receive charity. Besides it would be very harsh that people of sensitive feelings, and those who had moved in a better circle of society, should be deprived of relief till they were willing to have it at the expense of a public exposure of the circumstance in the newspapers.

We give the following quotation, although we do not anticipate the calamity to which allusion is made.

"The tendency of the present age is a mania towards the establishment of charitable institutions. It runs riot in a wild undefined wish to do good. Such liberality has always been the most marked in the decay of nations. It is one of the unequivocal precursors of that decay, and tends powerfully to aggravate the evils which it attempts to correct."

The number of persons receiving gratuitous medical aid in Sheffield is about 6,000 annually. Sheffield we are told contains 100,000 persons, and is surrounded by a densely populated country, so that it may be fairly estimated that about one in twenty of the population is thus relieved. In London, containing a million and a half of people, about 250,000 receive gratuitous medical aid annually, which is one-sixth part of the population. Many persons, however, both here and in Sheffield, are no doubt entered more than once during the same year.

There is one evil of indiscriminate medical charity which is only hinted at in this volume, namely, its injury to the medical profession. The working classes are the natural support of the commencing practitioner, and afford him a means of introduction to more profitable practice. These, and even the class above them, too often flock to charities; and we know of no cause which has so powerfully contributed towards degrading the greater part of general practitioners in this metropolis into the condition of shopkeepers, as the boundless profusion of gratuitous medical relief, showered down generally on all who may ask, without the slightest inquiry.

Although we have had to express our dissent from some of the opinions delivered in this volume, we believe it will lead to a searching enquiry on the subject, and occasion the reform of abuses in the medical charities both at Sheffield and other places. We consider that the author (who has not prefixed his name to the work) has conferred a benefit on society by his labour.

